# Supplementary material for: Evaluation of symptomatology and viral load among residents and healthcare staff in long-term care facilities: A coronavirus disease 2019 retrospective case-cohort study
Source: PLoS One. 2022 Nov 3;17(11):e0276796. doi: 10.1371/journal.pone.0276796 (PMC9632776; doi:10.1371/journal.pone.0276796)
Supplement: S1 Table — a) Sex was unknown for 6 residents. b) Age was unknown for 2 staff and 4 residents. c) α ≤ 0.01. d) Comorbidities were unknown for 554 COVID-19- residents, and 24 COVID-19+ residents, for a total of 578 residents. e) α ≤ 0.001. (DOCX) [file pone.0276796.s001.docx]

# **Supporting information**

**Supplemental table 1 (S1): Descriptive statistics of staff and resident characteristics by test result.**

|  | **LTCF staff (n=1,457)** | | | | **LTCF residents (n=1,540)** | | | |
| --- | --- | --- | --- | --- | --- | --- | --- | --- |
|  | **COVID-19- (n=945)** | | **COVID-19+ (n=512)** | | **COVID-19- (n=844)** | | **COVID-19+ (n=696)** | |
|  | n/N | % | n/N | % | n/N | % | n/N | % |
| Sex: female | 852/945 | 90.2 | 453/512 | 88.5 | 584/839^a^ | 69.6 | 480/695^a^ | 69.1 |
| Age in years (mean, standard deviation)^b^ | 41.2, 13.7^c^ |  | 43.5, 13.7^c^ |  | 83.7, 9.3 |  | 84.1, 8.6 |  |
| Comorbidity^d^ |  |  |  |  |  |  |  |  |
| Yes | 155/945 | 16.4 | 77/512 | 15.0 | 47/290^e^ | 16.2 | 545/672^e^ | 81.1 |
| Pregnant | 8/945 | 0.8 | 3/512 | 0.6 | 0/290 | 0.0 | 0/672 | 0.0 |
| Cardiovascular disease | 43/945 | 4.6 | 27/512 | 5.3 | 19/290^e^ | 6.6 | 413/672^e^ | 61.5 |
| Diabetes | 20/945 | 2.1 | 4/512 | 0.8 | 7/290^e^ | 2.4 | 143/672^e^ | 21.3 |
| Liver disease | 3/945 | 0.3 | 1/512 | 0.2 | 0/290 | 0.0 | 6/672 | 0.9 |
| Chronic neurological/neuromuscular disease | 8/945 | 0.8 | 2/512 | 0.4 | 2/290^e^ | 0.7 | 90/672^e^ | 13.4 |
| Immunodeficiency | 5/945 | 0.5 | 5/512 | 1.0 | 0/290 | 0.0 | 1/672 | 0.1 |
| Kidney disease | 2/945 | 0.2 | 4/512 | 0.8 | 1/290^e^ | 0.3 | 84/672^e^ | 12.5 |
| Chronic Pulmonary Disease | 64/945 | 6.8 | 30/512 | 5.9 | 14/290^e^ | 1.7 | 97/672^e^ | 13.9 |
| Malignancy | 6/945 | 0.6 | 3/512 | 0.6 | 10/290 | 3.4 | 36/672 | 5.4 |
| Obesity | 11/945 | 1.2 | 7/512 | 1.4 | 0/290^c^ | 0.0 | 17/672^c^ | 2.5 |
| Dementia/Alzheimer | 0/945 | 0.0 | 0/512 | 0.0 | 16/290^e^ | 5.5 | 261/672^e^ | 38.8 |
| Parkinson | 0/945 | 0.0 | 0/512 | 0.0 | 0/290^c^ | 0.0 | 14/672^c^ | 2.0 |
| Notes:   1. *Sex was unknown for 6 residents.* 2. *Age* *was unknown for 2 staff and 4 residents.* 3. *α ≤ 0.01.* 4. *Comorbidities were unknown for 554 COVID-19- residents, and 24 COVID-19+ residents, for a total of 578 residents.* 5. *α ≤ 0.001.* | | | | | | |  | |
