# Supplementary material for: Evaluation of symptomatology and viral load among residents and healthcare staff in long-term care facilities: A coronavirus disease 2019 retrospective case-cohort study
Source: PLoS One. 2022 Nov 3;17(11):e0276796. doi: 10.1371/journal.pone.0276796 (PMC9632776; doi:10.1371/journal.pone.0276796)
Supplement: S2 Table — (DOCX) [file pone.0276796.s002.docx]

# **Supporting information**

**Supplemental table 2 (S2): Descriptive statistics of staff (n=4) and resident (n=163) characteristics with unknown COVID-19 test result.**

|  | **LTCF staff (n=4)** | | **LTCF residents (n=163)** | |
| --- | --- | --- | --- | --- |
|  | n/N | % | n/N | % |
| Sex |  |  |  |  |
| Female | 3/4 | 75 | 81/163 | 49.7 |
| Age in years (mean, standard deviation) | 39.3, 18.0 |  | 84.7, 7.7 |  |
| Comorbidity |  |  |  |  |
| Yes | 0/4 | 0.0 | 157/163 | 96.3 |
| Pregnant | 0/4 | 0.0 | 0/163 | 0.0 |
| Cardiovascular disease | 0/4 | 0.0 | 92/163 | 56.4 |
| Diabetes | 0/4 | 0.0 | 31/163 | 19.0 |
| Liver disease | 0/4 | 0.0 | 1/163 | 0.6 |
| Chronic neurological/neuromuscular disease | 0/4 | 0.0 | 36/163 | 22.1 |
| Immunodeficiency | 0/4 | 0.0 | 0/163 | 0.0 |
| Kidney disease | 0/4 | 0.0 | 15/163 | 9.2 |
| Chronic Pulmonary Disease | 0/4 | 0.0 | 27/163 | 16.6 |
| Malignancy | 0/4 | 0.0 | 14/163 | 8.6 |
| Obesity | 0/4 | 0.0 | 3/163 | 1.8 |
| Dementia/Alzheimer | 0/4 | 0.0 | 120/163 | 73.6 |
| Parkinson | 0/4 | 0.0 | 8/163 | 4.9 |
| Fatality rate | 0/4 | 0.0 | 162/163 | 99.4 |
