# Supplementary material for: Evaluation of symptomatology and viral load among residents and healthcare staff in long-term care facilities: A coronavirus disease 2019 retrospective case-cohort study
Source: PLoS One. 2022 Nov 3;17(11):e0276796. doi: 10.1371/journal.pone.0276796 (PMC9632776; doi:10.1371/journal.pone.0276796)
Supplement: Attachment — Submitted filename: Response to Reviewers.docx [file pone.0276796.s003.docx]

# Comments from the editor

**Editor comment 1:** Please ensure that your manuscript meets PLOS ONE's style requirements, including those for file naming. The PLOS ONE style templates can be found at 
<https://journals.plos.org/plosone/s/file?id=wjVg/PLOSOne_formatting_sample_main_body.pdf> and 
<https://journals.plos.org/plosone/s/file?id=ba62/PLOSOne_formatting_sample_title_authors_affiliations.pdf>

*We have revised our manuscript to comply with PLOS ONE’s style requirements.*

**Editor comment 2:** During your revisions, please confirm whether the wording in the title is correct and update it in the manuscript file and online submission information if needed. Specifically, we believe that "coronavirus-19" should be corrected to either "COVID-19" or "coronavirus disease 2019".

*Thank you for pointing this out. We have adjusted the title to: “Evaluation of symptomatology and viral load among residents and healthcare staff in long-term care facilities: a coronavirus disease 2019 retrospective case-cohort study”.*

**Editor comment 3:** We note that you have stated that you will provide repository information for your data at acceptance. Should your manuscript be accepted for publication, we will hold it until you provide the relevant accession numbers or DOIs necessary to access your data. If you wish to make changes to your Data Availability statement, please describe these changes in your cover letter and we will update your Data Availability statement to reflect the information you provide.

*The data is already made publicly accessible to registered users of Synapse at: https://doi.org/10.7303/syn26939574.*

**Editor comment 4:** Your ethics statement should only appear in the Methods section of your manuscript. If your ethics statement is written in any section besides the Methods, please move it to the Methods section and delete it from any other section. Please ensure that your ethics statement is included in your manuscript, as the ethics statement entered into the online submission form will not be published alongside your manuscript.

*The ethics statement is now included at the end of the methods section under the heading ‘Declarations’. Additionally, because our ethical statement required clarification, we have pre-emptively adjusted our statement:*

*“The Medical Ethics Committee of Maastricht University Medical Centre (MUMC+) exempted this study from official approval under prevailing laws in the Netherlands after official review (METC number: 2021-2901).” (lines 213-216)*

**Editor comment 5:** Please include captions for your Supporting Information files at the end of your manuscript, and update any in-text citations to match accordingly. Please see our Supporting Information guidelines for more information: <http://journals.plos.org/plosone/s/supporting-information>.

*We have revised our manuscript to comply with PLOS ONE’s supporting information guidelines.*

# Comments from reviewer #1

*Reviewer one addresses three major issues, as well as several minor comments throughout the manuscript. We would like to address each of their major points first, after which we will address each of their minor points. We will refer to the line numbers of the revised manuscript for any changes made.*

**Reviewer 1 Major Comment 1:** The distribution of symptoms in the comparison groups (test-negative staff and residents) is likely to depend on the testing strategy, therefore the association of symptoms with test-positivity may also depend on the testing strategy. In order to investigate this potential effect, the authors should consider 1) adding a variable with the three different testing policy periods (27 Feb-5 April/6 April-16 April/17 April-1 June) in the multiple logistic regression model (and also test for interaction with this variable), and; 2) as testing policies are not necessarily applied in the same way in each LTCF, add an LTCF variable, either as fixed effect or as random effect in a mixed effects model.

*We thank the reviewer for their valuable comment and agree with this major comment. As such, we ran the multivariable model with an added variable accounting for the testing policy periods. For staff, this resulted in no significant interactions, but the variable for testing policy periods was significant on its own for female staff (aOR 0.67 (95CI 0.46-0.97)); women had higher odds to test negatively for COVID-19 later on in the study period. This is likely due to an increase in (preventive) testing among healthcare workers who experienced a hint of possible symptoms.*

*However, for residents, the variable for testing policy periods had a significantly higher OR, meaning both sexes had higher odds of having COVID-19 later on in the pandemic (aOR 3.00 (95CI 1.13-7.97) for men, and aOR 4.61 (95CI 2.41-8.82) for women). This is likely due to the continued spread and prevalence of the virus among LTCF residents across time. Furthermore, there was interaction among testing policy period and fever, and testing policy period and cough or female residents. Therefore, we also checked how the ORs would change if we stratified for testing period. Fortunately, the interactions for cough and fever are minor and do not alter the direction of the ORs for fever and cough in our main analyses. As such, we have chosen to not stratify further based on these symptoms. However, we did add the following to the Methods section:*

*“Furthermore, because testing policy changed during our study’s time period, the composition of included staff and residents may have influenced the ORs of the included symptoms. To assess the effect which testing policy may have had on the composition of included residents and staff, we also conducted a multivariable model with an added variable for testing policy periods (27 Feb - 5 April, 6 April-16 April, 17 April-1 June). The interactions between testing policy period and the symptoms were limited and did not influence our found effects for symptoms and COVID-19.” (lines 196-202)*

*Additionally, we feel that we should also add this as a part of our limitations section, which resulted in the following being added to the limitations section of the manuscript:*

*“Furthermore, because little was known of the SARS-CoV-2 virus, guidelines and measures were adapted as new information became available. This resulted in the suspect case definition for COVID-19 to change during the study period. Symptoms besides fever, dyspnoea, and cough, were not included in the initial suspect case definition. Although, the effects of these symptom do not seem to influence our findings when examined for each testing policy period separately, one should take into account the overrepresentation of symptoms such as fever, dyspnoea, and cough.” (lines 387-393)*

*Additionally, while it is possible that different LTCFs had different testing policies, we are unable to correct for this. Because these data were gathered in a hectic time period, the associated LTCF for each case was often not registered or registered in a different way between data managers. As such, we have decided to exclude these data, as it is highly likely that this data is of insufficient quality. However, we do agree with the reviewer that this is a limitation of our study. As a result, we have added the following to the limitations section of this manuscript:*

*“By requesting LTCFs to make an inventory of deaths who were suspect of COVID-19, we were able to estimate not lab-confirmed COVID-19 related deaths. These suspected cases had symptoms suggestive of COVID-19, but did not have a lab-confirmed test, and were reported to the PHS on physician judgement based on the clinical picture. Additionally, because these data were gathered within a period of (inter)national crisis, data gathering was not as streamlined as compared to prepared studies. As a result, data with regards to which case belonged to which LTCF was unable to be included in our dataset. Therefore, we could not correct for LTCF specific testing policies. This also means that we could not compare the number of included residents to the total number of residents (beds) and staff.” (lines 378-387)*

**Reviewer 1 Major Comment 2:** The completeness of data on symptoms is very different between staff and residents and between test-negatives and test-positives. While for staff, symptoms were unknown for only 48 (5.1%) covid-19 negatives and 8 (1.6%) covid-19 positives, in residents the number of unknowns was much higher with 623 (73.8%) and 141 (20.3%), respectively. This differential distribution of missing values may of course also affect the observed associations. In the discussion the authors say “Due to the symptom-based testing policy at that time, we did not test for asymptomatic patients.” , so missing data on symptoms would not be due to testing of asymptomatic residents (in e.g. “vulnerable groups”)? On L89, the authors report for the period since 17 April “Although negatively tested cases were also reported to the PHS by the LTCFs, no additional information on comorbidities or symptoms was provided by the LTCFs.” Doesn’t this mean that the analysis of symptoms should be restricted to the period before 17 April?

*We agree with the reviewer on this point that only cases should be analysed for whom we have complete symptom information. This does not mean however that the analyses should be restricted to the time period before April 17^th^; analyses should be possible as long as we have all information available. To better reflect that we only included residents and staff of whom we have complete symptom data, we have adjusted our table titles to include ‘with known symptoms and test results’.*

**Reviewer 1 Major Comment 3:** It is unclear why most analyses were stratified by sex. Was this because a statistically significant interaction with sex was observed when pooling the data? If so, this should be mentioned. On the one hand, stratifying reduces statistical power (especially for male staff and residents) and on the other hand multiplying the number of analysed associations by two increases the likelihood of finding statistically significant results by chance. In several cases, the difference in aORs between sexes seems to be not significant. Is the stratification by gender always justified?

*At the time, there was a need for sex-specific information with regards to COVID-19. As such, we paid particular attention to the difference in men and women, by creating a model for each sex.*

*To reinforce our decision to stratify by sex, early on, it became clear that the case fatality rate was higher among men compared to women. As a result, if we wouldn’t stratify for sex, the increase in CFR among women would disappear in our multinominal regression analysis: the effect among women would be cancelled out with the inclusion of men.*

*We do agree that the manuscript does not clarify our reason for stratifying well enough. As such, we have added the following:*

*“Because several calls urged for sex-specific information in COVID-19 studies, as well as an increased risk of mortality for male patients with COVID-19, analyses were stratified by sex [36, 37].” (lines 164-166)*

Another point, the lower Ct values found in male compared to female patients is an observation that is not very consistent in literature and this should be better addressed in the discussion. The higher mortality in males is quite consistent though (and not only for COVID-19).

*We agree with the reviewer with regards to the discussion surrounding Ct-values in men and women. Therefore, we have added an additional paragraph to the discussion:*

*“With regards to a difference in viral load level between men and women, the literature seems unclear. Several studies reported varying viral load levels when comparing men and women; some reported women had higher load levels and some reported lower load levels compared to men, as well as studies reporting no difference in viral load level [56-58]. However, there seems to be support for an overall lower viral load level and COVID-19 outcomes for women, as the immune regulatory genes from the X chromosomes in women appear to cause lower viral load levels, inflammation and death after COVID-19 infections [57, 59].” (lines 350-356)*

Furthermore, please have the manuscript reviewed by a native speaker as there is some room for improvement of the English language.

*We have had several colleagues read and made improvements to the overall quality of English throughout the manuscript.*

**Reviewer 1 Minor Comment 1:** L37. Therefore, physicians should consider testing residents even when only aspecific symptoms are present.
⎝ Actually, the ECDC testing guidance recommended to test all residents and staff (symptomatic or not) once there was/is one confirmed case of COVID-19 in the LTCF.

*Regular testing was indeed recommended by the ECDC. However, at the time, LTCFs did not always have the resources to (preventively) test for COVID-19 among all residents and staff. We wanted to emphasise that in a scenario where not everyone can get tested,* *testing priority should go to residents who (only) show aspecific symptoms. In order to further clarify this, we have adjusted the abstract to the following:*

*“Therefore, physicians should consider giving testing priority to residents who (only) show aspecific symptoms whenever available resources prohibit testing of all residents.” (lines 41-42)*

**Reviewer 1 Minor Comment 2:** L 48. the proportion of COVID-19-related deaths still ranges between 0% and 62% in LTCF residents in European countries, as reported up until November 9th 2021 [8].
⎝ Reference 8 was updated on 21 Feb 2022 and doesn’t show these data anymore.

*We thank the reviewer for this comment. We have added a new hyperlink to this reference which should now show the table discussed in the introduction.*

**Reviewer 1 Minor Comment 3:** L 51. LTCF residents often have chronic diseases, are mentally incapable and have complex health needs
⎝ Suggest “LTCF residents often have chronic diseases, mental impairment and complex health needs”
(the “often” should apply to all three conditions)

*This has been adjusted according to the reviewer’s feedback.*

**Reviewer 1 Minor Comment 4:** L71. During the first wave of the COVID-19 pandemic, almost all (suspect) COVID-19 cases for residents and staff were tested by the Public Health Services (PHS) in the South Limburg region. Additionally, all LTCF tests not performed by our PHS were mandatory notified. With this virtually fully complete LTCF dataset of the classic SARS-CoV-2 variant within one region, we evaluated which symptoms are associated with COVID-19 in LTCF staff and residents, as well as assessed the relationship of viral load with symptoms and case fatality rate (CFR).
⎝ Most of this is repeated afterwards (e.g. L85 and following. L 105). Suggest to replace with “The objectives of our study were to assess symptoms associated with COVID-19 in LTCF staff and residents and to assess the relationship between viral load and COVID-19-related symptoms and case fatality, in LTCF residents and staff in the South Limburg region.”

*This has been adjusted according to the reviewer’s feedback.*

**Reviewer 1 Minor Comment 5:** L73. Additionally, all LTCF tests not performed by our PHS were mandatory notified.
⎝ PCR tests or rapid antigen tests ? If RADTs, were they confirmed by PCR?

*All LTCF tests were PCR tests, of which the results were communicated to the PHS. However, this part is not included anymore, as this section has been omitted from the manuscript per reviewers’ feedback (see Minor Comment 4).*

**Reviewer 1 Minor Comment 6:** ⎝ “mandatorily” rather than mandatory

*This is not applicable anymore, as this section has been omitted from the manuscript per reviewer feedback (see Minor Comment 4).*

**Reviewer 1 Minor Comment 7:** L77. Our assessment of the first COVID-19 wave in LTCFs in the Netherlands has contributed to the limited research on Ct-values as an indicator of viral load and it’s relation with COVID-19 symptomatology and mortality.
⎝ Delete sentence (or move to conclusion)

*We respectfully disagree with the reviewer in deleting or moving this part. As per PLOS ONE’s author guidelines, the introduction should ‘conclude with a brief statement of the overall aim of the work and a comment about whether that aim was achieved.’ However, should the editor agree with the reviewer, we would suggest moving this section to the conclusion section of our manuscript.*

**Reviewer 1 Minor Comment 8:** L 82. We performed a cross-sectional epidemiological and laboratory analysis of LTCF residents and staff who were tested for COVID-19 in South Limburg, the Netherlands, from February 27th 2020 to June 1st 2020.
⎝ Not cross-sectional, rephrase. The title mentions cohort study, but it’s not a real cohort study either as non-tested staff and residents are not included.

*We agree with this comment. We have removed ‘cross-sectional’ from this line, rephrasing it to:*

*“We performed an epidemiological and laboratory analysis of LTCF residents and staff who were tested for COVID-19 in South Limburg, the Netherlands, from February 27^th^ 2020 to June 1^st^ 2020.” (lines 82-84)*

**Reviewer 1 Minor Comment 9:** Title should be adapted

*We have rephrased the title of the manuscript to:*

*“Evaluation of symptomatology and viral load among residents and healthcare staff in long-term care facilities: a coronavirus disease 2019 retrospective case-cohort study” (lines 1-4)*

**Reviewer 1 Minor Comment 10:** ⎝ How many LTCFs were included?

*Unfortunately, we cannot say how many LTCFs were included. About 109 LTCFs are known to our PHS region, of which the majority has provided information on their COVID-19 testing. However, the names of included LTCFs were often not registered, or were registered in different ways, which made it hard to allocate all cases to a certain LTCF. As such, this information was, regretfully, not included in the final dataset.*

**Reviewer 1 Minor Comment 11:** L96. In response to the increase of COVID-19 cases within LTCFs, additional infection control measures were implemented by the LTCFs, among which a (temporarily) halt of admission of new residents and a limited number of visitors, or total visitor ban. LTCF staff wore gowns, surgical mouth masks, and gloves when they expected to get within 1.5 meters of COVID-19 positive residents or their surroundings.
⎝ … (temporary) admission stop , limitation of the number of visitors or a total visitor ban, depending on the LTCF (?)

*A nationwide halt on LTCF visitors was implemented from the 20^th^ of March. Admission stops were dependent on each LTCF, for example dependent on the presence (yes/no) of a COVID-19 cluster, but were never introduced as a national COVID-19 control measure.*

**Reviewer 1 Minor Comment 12:** ⎝ surgical mouth masks -> medical face masks

*This has been adjusted according to the reviewer’s feedback.*

**Reviewer 1 Minor Comment 13:** L 108. Additionally, a suspected case should have a history of travel or residence in a country/area reporting local or community transmission, or have had to be in close contact with a confirmed, or probable COVID-19 case in the past two weeks [33, 34].
⎝ Was South Limburg was considered an area reporting community transmission at the time? If so, from when?

*South Limburg was not considered an area reporting community transmission at the time.*

**Reviewer 1 Minor Comment 14:** L111. As of April 6th 2020, testing policy was expanded. Whereas testing was first reserved to cases reporting serious health symptoms, tests were now also performed within vulnerable groups when this was necessary to be able to provide care or treatment. Tests were also available for healthcare staff when they showed symptoms indicative of COVID-19, and when they were essential in providing care. As of April 17th, LTCFs had the option of performing COVID-19 tests on residents themselves.
⎝ The change of testing policy on 6 April should be mentioned in the section “Study design, setting and test policy”

*This has been adjusted according to the reviewer’s feedback.*

**Reviewer 1 Minor Comment 15:** ⎝ What do you mean with “vulnerable groups” in the context of an LTCF? Aren’t all residents vulnerable because of their age? So there was never a policy of testing all residents from February 27th 2020 to June 1st 2020?

*At the time, vulnerable groups were identified as groups of people who have a higher risk of severe COVID-19 outcomes. These were identified as people aged 70 and older as well as people on the basis of underlying disease. As such, almost all LTCF residents were considered to be part of this ‘vulnerable group’.*

*In terms of testing policy, there was a nationwide testing policy for testing residents in LTCFs. Until June 1, this policy was aimed at determining whether COVID-19 had been introduced in a LTCF ward, so that infection-preventive measures could be taken. If one or two residents in a ward tested positive, the introduction of the SARS-CoV-2 virus was established and it was assumed that other residents in the ward with similar complaints also had COVID-19. However, they did not need to be tested. Health care professionals were only tested if they showed signs of disease. As of June 1^st^, everyone with symptoms could register for a PCR test.*

*Because these ‘vulnerable groups’ were not clearly defined in our manuscript, we have added the following:*

*“As of April 6th 2020, testing policy was expanded to include vulnerable groups when testing was necessary to be able to provide care or treatment. At the time, vulnerable groups were identified as groups of people who had a higher risk of severe COVID-19 outcomes, which included people aged 70 and older, as well as people on the basis of underlying disease. Tests were also available for healthcare staff when they showed symptoms indicative of COVID-19, which at the time included symptoms of a cold, such as a runny nose, sneezing, sore throat, cough, shortness of breath, subfebrility or fever, and sudden loss of smell and/or taste. Staff were only eligible for testing if they were also essential in providing care [33].” (lines 87-95)*

**Reviewer 1 Minor Comment 16:** ⎝ The case definition as such is actually not clearly mentioned in this section

*We agree that the suspect case definition could be described more accurately. As such, we have adjusted this part of the methods section as followed:*

*“At this point in time, the suspect case definition included a sudden onset of fever (38 degrees Celsius), paired with respiratory symptoms like cough or shortness of breath. In addition to the aforementioned symptoms, a suspected case should have a history of travel or residence in a country/area reporting local or community transmission (defined through a large number of cases which cannot be linked to transmission chains or multiple unrelated clusters in several areas of the country/area), or have had to be in close contact with a confirmed, or probable COVID-19 case in the past two weeks [34].” (lines 119-125)*

**Reviewer 1 Minor Comment 17:** L193. When we included the highly suspected COVID-19 deaths, the overall CFR increased to 40.4% (347/859), of which 53.6% (159/297) among men and 33.5% (188/561) among women.
⎝ What is the denominator here exactly (n=859)?

*N=859 for all cases, so a total CFR of 40.4%. Of the 859 cases, 297 were men, and 561 were women, and 1 had no registered sex. The CFR of men was 53.6% (159 died out of the 297 positive men), and the CFR for women was 33.5% (188 women died out of the 561 positive women).*

**Reviewer 1 Minor Comment 18:** ⎝ In addition, it would be important to know the total population of residents and staff in the included LTCFs for the study period (tested and non-tested)? Alternatively for residents, the number of beds in the included LTCFs. Also the number of LTCFs seems to be missing.

*Unfortunately, these data could not be included, as we are not certain which LTCFs have been included (has been addressed in Major Comment 1).*

**Reviewer 1 Minor Comment 19:** L200. Multivariate -> multiple or multivariable

*This has been adjusted according to the reviewer’s feedback.*

**Reviewer 1 Minor Comment 20:** L209-211: What does the Note a (“Unless stated otherwise”) mean?

*This was added to indicate missing data whenever the number of men and women did not add up to the total number of included cases. However, these tables have since been revised and this note has been omitted from the manuscript.*

**Reviewer 1 Minor Comment 21:** Note c in Table 1a and note d in Table 1b on the "creation of an artificial case" is unclear

*We agree that the note regarding the creation of an artificial case was too short and unclear. As such we have rephrased this note and added the following section to the methods section of the manuscript:*

*“For some symptoms, it was not possible to calculate the odds ratio, because there would not be a case with a certain symptom in either the COVID-19 negative, or the COVID-19 positive group. As such, in order to give some interpretation to the odds ratio, we imputed a temporary case. Aside from the relevant symptom, this temporary case was assigned average values for other variables such as age to limit its influence on other variables.” (lines 190-195)*

**Reviewer 1 Minor Comment 22:** L237-L242: suggest to remove the line Symptoms “yes” from the Tables, that’s implicit

*This has been adjusted according to the reviewer’s feedback.*

**Reviewer 1 Minor Comment 23:** See general comments, which may have an impact on the discussion. The study limitations section should be further developed

*We have taken note of the reviewer’s comments and have added several limitations which previously were not adequately discussed. As such, the following has been added:*

*“Additionally, because these data were gathered within a period of (inter)national crisis, data gathering was not as streamlined as compared to prepared studies. As a result, data with regards to which case belonged to which LTCF was unable to be included in our dataset. Therefore, we could not correct for LTCF specific testing policies. This also means that we could not compare the number of included residents to the total number of residents (beds) and staff.” (lines 382-387)*

**Reviewer 1 Minor Comment 24:** L342. By requesting LTCFs to make an inventory of deaths who were suspect of COVID-19, we were able to estimate a portion of asymptomatic COVID-19 related deaths.
⎝How can suspected COVID-19-related deaths (I assume not lab-confirmed but with a clinical picture compatible with COVID-19) be asymptomatic?

*The reviewer is correct.* *These cases were not asymptomatic, but had symptoms suggestive of COVID-19 and no lab-confirmed test. These cases were reported to the PHS on physician judgement based on the clinical picture. We have added the following to clarify this:*

*“By requesting LTCFs to make an inventory of deaths who were suspect of COVID-19, we were able to estimate not lab-confirmed COVID-19 related deaths. These suspected cases had symptoms suggestive of COVID-19, but did not have a lab-confirmed test, and were reported to the PHS on physician judgement based on the clinical picture.” (lines 378-381)*

**Reviewer 1 Minor Comment 25:** L353. However, the variance in Ct-value between variants is limited, therefore upholding the findings of this study [56].
⎝Evidence regarding variant-dependent viral load is still evolving (e.g. Omicron), so this statement is too strong.

*We agree with the reviewer that this statement is too strong. Therefore, we have changed it to:*

*“Although Ct-values are known to differ per virus variant, this variance seems to be limited. Therefore, it is likely that the findings of this study will be in line with other (future) variants of the SARS-CoV-2 virus [60]. However, future studies are needed to verify this.” (lines 399-402)*

**Reviewer 1 Minor Comment 26:** ⎝Also the influence of vaccination and previous infection should be mentioned, not only regarding Ct values/viral load but also regarding symptoms.

*We agree with the reviewer that this wasn’t addressed. As such, we have added the following:*

*“**Furthermore, the effect of vaccination and previous infection may influence the viral load, as well as symptoms, which a person may have in COVID-19 infections following the first COVID-19 wave.” (lines 402-404)*

# Comments from reviewer #2

**Reviewer 2 Major Comment 1:** The authors stratify the results by sex throughout the manuscript and this is somewhat an odd choice since no significant differences between males and females were observed (all 95%CIs overlap). The number of significantly COVID-19 associated symptoms differs by sex, but this seems to be a sample number issue due to the lower number of male residents and male staff members. The authors mention in the results a significant difference in mortality between males and females, but do not show these results in the presented tables.

*At the time, there was a need for sex-specific data with regards to COVID-19. As such, we paid particular attention to the difference in men and women by creating a model for each sex. Early on, it became clear that the case fatality rate was higher among men compared to women. As a result, if we wouldn’t stratify for sex, the increase in CFR among women would disappear in our multinominal regression analysis: the effect among women would be cancelled out with the inclusion of men. These CFR data are not included in table S1 or S2, but are discussed in the first paragraph of the results section of the manuscript. However, we think the reviewer expected these data to be included in the table, because the sentence following our section about the CFR refers to table S2. Therefore, we have restructured this section of the results to better reflect which data are included in the tables. However, should the editor request us to provide these data in the table, we would be happy to do so.*

*To summarize, we do agree that the manuscript does not clarify our reason for stratifying well enough. As such, we have added the following:*

*“Because several calls urged for sex-specific information in COVID-19 studies, as well as an increased risk of mortality for male patients with COVID-19, analyses were stratified by sex [36, 37].” (lines 164-166)*

**Reviewer 2 Major Comment 2:** A major concern is that the case definition changed over the course of the study and that the study outcomes (i.e. fever and respiratory symptoms) were part of the case definition (at least in the first phase). This makes the presented ORs between the cases and controls difficult to interpret. I.e. cases with subfebrility could not be found in the first phase since those were excluded based on the case definition. The authors do not discuss this limitation in the discussion.

*We thank the reviewer for this critical remark. Whilst we do agree with the reviewer that this is a limitation to the study, it also is a representation of the time period in which this study has been conducted, in which* *little was known of the SARS-CoV-2 virus and guidelines and measures were adapted as new information became available.*

*To examine the possible effect of a change in testing policy, we ran the multivariable model with an added variable accounting for the testing policy periods. For staff, this resulted in no significant interactions, but the variable for testing policy periods was significant on its own for female staff (aOR 0.67 (95CI 0.46-0.97)); women had higher odds to test negatively for COVID-19 later on in the study period. This is likely due to an increase in (preventive) testing among healthcare workers who experienced a hint of possible symptoms.*

*However, for residents, the variable for testing policy periods had a significantly higher OR, meaning both sexes had higher odds of having COVID-19 later on in the pandemic (aOR 3.00 (95CI 1.13-7.97) for men, and aOR 4.61 (95CI 2.41-8.82) for women). This is likely due to the continued spread and prevalence of the virus among LTCF residents across time. Furthermore, there was interaction among testing policy period and fever, and testing policy period and cough or female residents. Therefore, we also checked how the ORs would change if we stratified for testing period. Fortunately, the interactions for cough and fever are minor and do not alter the direction of the ORs for fever and cough in our main analyses. As such, we have chosen to not stratify further based on these symptoms. However, we did add the following to the Methods section:*

*“Furthermore, because testing policy changed during our study’s time period, the composition of included staff and residents may have influenced the ORs of the included symptoms. To assess the effect which testing policy may have had on the composition of included residents and staff, we also conducted a multivariable model with an added variable for testing policy periods (27 Feb - 5 April, 6 April-16 April, 17 April-1 June). The interactions between testing policy period and the symptoms were limited and did not influence our found effects for symptoms and COVID-19.” (lines 196-202)*

*Additionally, like the reviewer mentioned, we feel that we should also add this as a part of our limitations section, which resulted in the following being added to the limitations section of the manuscript:*

*“Furthermore, because little was known of the SARS-CoV-2 virus, guidelines and measures were adapted as new information became available. This resulted in the suspect case definition for COVID-19 to change during the study period. Symptoms besides fever, dyspnoea, and cough, were not included in the initial suspect case definition. Although, the effects of these symptom do not seem to influence our findings when examined for each testing policy period separately, one should take into account the overrepresentation of symptoms such as fever, dyspnoea, and cough.” (lines 387-393)*

**Reviewer 2 Major Comment 3:** The authors used the forced entry model selection method for the multivariable logistic regression model and included all variables from the univariate analysis with a p-value above 0.1 to the model. Although multiple variable selection methods are used in the field, this does not seem to be the best choice. Multiple variables were added to the model which do not significantly contribute to the model (based on the provided information), and make the model unnecessary complex. It might be better to use a mixture of forward and reverse variable selection together with AIC or BIC. Also: two different models were used for males and females (i.e. the model in table 1 shows age + 3 variables for males and age + 5 variables for females), raising questions whether both models can be compared.

*We agree with the reviewer that it is not completely correct to compare models whenever they do not have the same included variables. Therefore, we have added the symptoms which were significant at the univariable analysis for both men and women to the multivariable model in order for the models to be comparable. Additionally, we have redone our analyses with an a of alpha of 0.05 instead of 0.1. We agree that a forward or backward selection strategy would be both be a good option. However, because we use univariable analyses to select symptoms for the multivariable analysis, we feel that the forced entry method is also justifiable.*

**Reviewer 2 Minor Comment 1:** Line 3: use official nomenclature throughout the manuscript: “COVID-19” for the disease and “SARS-CoV-2” for the virus.

*We thank the Reviewer for pointing this out to us. Cases in which COVID-19 was not used in the nomenclature way have been adjusted.*

**Reviewer 2 Minor Comment 2:** Line 3: the term cohort study is misused here in my opinion.

*The term ‘cohort’ has been removed.*

**Reviewer 2 Minor Comment 3:** Line 23: write “LTCF” out in full when first used in the abstract

*This has been adjusted according to the reviewer’s feedback.*

**Reviewer 2 Minor Comment 4:** Line 46: I suggest to use “wildtype SARS-CoV-2 Wuhan strain” instead of “classic SARS-CoV-2 virus”.

*This has been adjusted according to the reviewer’s feedback.*

**Reviewer 2 Minor Comment 5:** Line 50: “up” can be omitted. I suggest to add the reporting organisation (European Centre for Disease Prevention and Control) to the sentence.

*This has been adjusted according to the reviewer’s feedback.*

**Reviewer 2 Minor Comment 6:** Line 52-59: these sentences need to be rephrased and can be shortened since they convey the same message twice. Also: rapid antigen assays and availability of RT-PCR testing have made it easier to recognize COVID-19 outbreaks in LTCFs.

*This has been adjusted according to the reviewer’s feedback.*

**Reviewer 2 Minor Comment 7:** Line 86: please describe what “information about the person to be tested” was collected by phone.

*We thank the reviewer for this comment. We agree that this part is too vague. As such we have elaborated which information was gathered:*

*“Prior to the test, information about the person to be tested was gathered by phone, including date of birth, sex, symptoms, comorbidities, date of onset, profession, and whether they were a LTCF resident* *or LTCF staff member.” (lines 97-99)*

**Reviewer 2 Minor Comment 8:** Line 112: what is meant by serious health symptoms?

*Serious health symptoms refers to people with symptoms worse enough for them to be admittable to the regional hospital. In other cases, a patient’s general physician can request a test when the situation warrants one. To clarify this, the following has been added to the manuscript:*

*“**Testing was first reserved to cases reporting serious health symptoms, meaning whenever a case was sick enough to be admitted to the hospital, or when their general physician deemed a test to be necessary.” (lines 85-87)*

**Reviewer 2 Minor Comment 9:** Line 113: be specific. In which situations was it necessary to test vulnerable groups to be able to provide care or treatment? Did the LTCFs still use a case definition in this period?

*We agree that this part was not specific enough. Therefore, we have added the following:*

*“At the time, vulnerable groups were identified as groups of people who had a higher risk of severe COVID-19 outcomes, which included people aged 70 and older, as well as people on the basis of underlying disease.” (lines 89-91)*

**Reviewer 2 Minor Comment 10:** Line 114: which symptoms? Fever still included in the case definition?

*We thank the reviewer for their remark. We have added the following to clarify which symptoms were applicable for the testing criteria at the time:*

*“Tests were also available for healthcare staff when they showed symptoms indicative of COVID-19, which at the time included symptoms of a cold, such as a runny nose, sneezing, sore throat, cough, shortness of breath, subfebrility or fever, and sudden loss of smell and/or taste. Staff were only eligible for testing if they were also essential in providing care [33].” (lines 91-95)*

**Reviewer 2 Minor Comment 11:** Line 115-116: Did the LTCFs use a case definition in this period? What about screening of contacts of cases? I am asking since you barely have asymptomatic cases in the dataset.

*The case definition used in the LTCFs did not deviate from the nationwide case definition during our study period. However, residents were seen as a risk group (age 70+), which made them eligible for a test more quickly than the rest of the population. At the start of the pandemic, tests were only performed in cases with symptoms (fever with cough and/or dyspnea). This also applied for people who got into contact with a positive COVID-19 case, although these contacts had to be quarantined. Residents and staff without symptoms were only tested in exceptional cases where a test was deemed necessary for safely providing or receiving healthcare.*

**Reviewer 2 Minor Comment 12:** Line 169: I think the authors mean “forced entry method” instead of “forced enter method”

*This has been adjusted according to the reviewer’s feedback.*

**Reviewer 2 Minor Comment 13:** Line 259-260: In my view aspecific symptoms are by definition not discriminative.

*We agree with the reviewer and have replaced discriminative with ‘suggestive’.*

**Reviewer 2 Minor Comment 14:** Line 302: “original” can be omitted

*This has been adjusted according to the reviewer’s feedback.*

**Reviewer 2 Minor Comment 15:** Line 317 – 325: I suggest to omit “Although race…ethnic makeups” from the manuscript. This topic was not part of the study and statements on human race should be avoided in scientific publications.

*We agree with the reviewer and have decided to omit this part of the manuscript, as it does not add any additional information.*

**Reviewer 2 Minor Comment 16:** Line 326: “complete picture” sounds like an unusual statement for a scientist.

*We agree with the reviewer and have changed this sentence to the following:*

*“This study gave insights into COVID-19 testing within the Dutch LTCF setting in the first wave of the pandemic, by compiling a dataset of tested residents, as well as staff member from LTCFs in South Limburg.” (lines 362-364)*

**Reviewer 2 Minor Comment 17:** Line 326-337: this paragraph reads like it was written by the sales manager of the PHS.

*We agree that this paragraph may have come off too strongly. As such, we have made several changes throughout this paragraph:*

*“This study gave insights into COVID-19 testing within the Dutch LTCF setting in the first wave of the pandemic, by compiling a dataset of tested residents, as well as staff member from LTCFs in South Limburg. In doing so, we were able to compare a large number of negatively, as well as positively tested residents and staff who were symptomatic. Therefore, our findings are likely to be generalisable for LTCFs nationally, as well as internationally. By creating a link between epidemiological data and microbiological data, we were able to further examine CFR and viral load. Because all test results were determined by a single laboratory, no inter-lab variance could have taken place, which increases the comparability of the Ct-values. Additionally, intra-lab variance was limited by using the same technique and diagnostic device for all samples. Furthermore, by taking into the account the suspected deaths which have most likely been caused by COVID-19, we were able to estimate a more accurate representation of the CFR in this vulnerable population.” (lines 362-373)*

**Reviewer 2 Minor Comment 18:** Line 339: a few individuals without symptoms are in the dataset though?

*Some asymptomatic cases were included in the dataset because they were tested LTCF staff or residents within this study’s timeframe. However, these asymptomatic cases were tested as part of outbreak research performed by the PHS. As such, they should not have been included in the analyses. Therefore, we have redone our analyses without asymptomatic cases, as indicated by the revised tables and table titles.*

**Reviewer 2 Minor Comment 19:** Line 349-350: which role could Ct values have in the clinical decision making as reported by the referenced papers? And what does this study confirm or add to the existing literature?

*CT-values could provide insights into the infectiveness of a case, which could influence treatment decisions with regards to isolation. Our findings contribute to scientific knowledge with regards to the prognosis of outcomes of COVID-19 cases with low to high CT-values, and which symptoms might be associated with different CT-value levels.*

**Reviewer 2 Minor Comment 20:** Line 351-354: Contradictio in terminis: “Knowing that the Ct-value differs per virus variant” and “variance in Ct-value between variants is limited”. Does it differ or not? Or not yet a scientific consensus?

*We agree that this part needs some clarification. As such, we have added the following:*

*“**Although Ct-values are known to differ per virus variant, this variance seems to be limited. Therefore, it is likely that the findings of this study will be in line with other (future) variants of the SARS-CoV-2 virus [60]. However, future studies are needed to verify this.” (lines 399-402)*

**Reviewer 2 Minor Comment 21:** Line 534 ref 33: this is only a link to the general RIVM web archive website. Please add the information to the specific document.

*We thank the reviewer for this remark. We have decided to omit the reference to the RIVM web archive website, as it is impossible to create a link to the specific page; only a general link is available. We feel that the reference to the ECDC alone is enough to support this section of the manuscript.*

**Reviewer 2 Minor Comment 22:** Figure 2: Please add figure legend or describe the meaning of the colours in the figure description.

*We thank the reviewer for this comment. Whilst converting the image, the explanation for the colours was cut from the image. We have adjusted the image to include these data.*

**Reviewer 2 Minor Comment 23:** Tables: please describe or indicate the meaning of bold text.

*We thank the reviewer for this just remark. We have added the meaning of bold text to each of the tables.*

**Reviewer 2 Minor Comment 24:** Table 1B: Why is the unadjusted OR for female and fever not in bold? P-value above 0.1? Why added to the multivariable model?

*It was not significant at an alpha of 0.1, but because it was part of the ordinary variable for temperature increase in which subfebrility was significant, it was still included in the multivariable model.*

**Reviewer 2 Minor Comment 25:** Table 2a: Please add the notes in Table 2A. They cannot be similar to those in Table 2B.

*This has been adjusted according to the reviewer’s feedback.*
