# Supplementary material for: Evaluation of symptomatology and viral load among residents and healthcare staff in long-term care facilities: A coronavirus disease 2019 retrospective case-cohort study
Source: PLoS One. 2022 Nov 3;17(11):e0276796. doi: 10.1371/journal.pone.0276796 (PMC9632776; doi:10.1371/journal.pone.0276796)
Supplement: Attachment — Submitted filename: Response to Reviewers v2.docx [file pone.0276796.s004.docx]

# **Comments from reviewer #2**

*Reviewer two addresses three major issues, as well as several minor comments throughout the manuscript. We would like to address each of their major points first, after which we will address each of their minor points. We will refer to the line numbers of the revised manuscript for any changes made.*

**Reviewer 2 Major Comment 1:** Although significantly improved since the last submitted version, the description of the case definitions and testing policies can still be improved. I suggest to add one paragraph on the case definitions and testing guidelines and merge the respective sections from the paragraphs “Study design, setting and testing policy” and “Suspect case definition”. Please describe clearly how these different policies were applicable to LTCF residents (it seems like there were some overall National guidelines + some additional specific LTCF policies). Please add the policies for screening of LTCF residents who have had contact with a confirmed SARS-CoV-2 case (if those were included in the analysis) as well.

*We would like to thank the reviewer for once again taking their time to extensively review our work. Their comments are much appreciated.*

*We agree that the Materials and methods section of the manuscript required some restructuring. We have therefore decided to restructured this part of the manuscript. The paragraph ‘case definition and testing policy’ should better describe testing policy with regards to national guideline and as well as LTCF testing policy.*

*With regards to the testing of contacts, we have added the following:*

*“Contacts of a confirmed COVID-19 case were tested only when they were part of a vulnerable population group; people who had a higher risk of severe COVID-19 outcomes, such as people aged 70 and older, as well as people with underlying disease [33, 34].” (lines 89-91)*

**Reviewer 2 Major Comment 2:** It is sometimes challenging to understand what the authors mean since the English is sometimes weak or is not always written in scientific language. I feel it is not my job to correct for this and I strongly suggest to seek support if this knowledge is not available among the authors. I made some comments and suggestions to improve (see minor comments below), but would like to stress here that the manuscript requires an additional thorough editing to make it readable.

*We thank the reviewer for their suggestions to improve the level of English throughout the manuscript. For this revision of the manuscript, we have asked colleagues proficient in the English language to carefully review the manuscript. As a result, changes have been made throughout the manuscript.*

**Reviewer 2 Major Comment 3:** The methods are a bit messy with sometimes very long paragraphs. Statistical analysis is almost 2 pages long for example and contains details that may not be very important for the reader (i.e. how variables are named or coded).

*We agree with the reviewer that the structure of the methods can be improved. We have rearranged the structure, as well as reduced the length of the materials and methods section wherever possible. However, in order to be transparent in the assumptions and coding we have done, we feel that further reducing the size of this section would be detrimental to the manuscript’s overall quality.*

**Reviewer 2 Minor Comment 1:** Line 77 – 79: this sentence is redundant and can be omitted

*We respectfully disagree with the reviewer in deleting or moving this part. As per PLOS ONE’s author guidelines, the introduction should ‘conclude with a brief statement of the overall aim of the work and a comment about whether that aim was achieved.’ However, should the editor agree with the reviewer, we suggest moving this section to the conclusion section of our manuscript.*

**Reviewer 2 Minor Comment 2:** Line 85: Were all LTCF residents admitted to the hospital tested or was this indicated based on symptoms as well? Please clarify.

*This part of the materials and methods section describes the testing policy used for all residents of the Netherlands and not, as the reviewer suggests, testing policy specific to LTCF residents. Dutch residents were only tested for COVID-19 when they were deemed sick enough by the general practitioner, or when they were sick enough to be admitted to the hospital and displayed symptoms indicative of COVID-19, or unexplained pneumonia. LTCF residents were also only tested when they displayed severe symptoms indicative of COVID-19. However, as of April 6th, LTCF residents aged 70 years and older who displayed symptoms (fever (38 degrees Celsius) and cough and/or dyspnoea) were tested for COVID-19 when this was necessary for their care or treatment, regardless of their level of illness.*

**Reviewer 2 Minor Comment 3:** Do you mean that all LTCF residents 70+ were regularly and routinely tested? Were symptoms included in this case definition? How frequently were the residents tested if symptoms were not included?

*We thank the reviewer for their comment. No exceptions were made from the nationwide testing policy with regards to LTCF residents, meaning they were only tested when they were deemed sick enough by the general practitioner, or when they were sick enough to be admitted to the hospital and displayed symptoms indicative of COVID-19, or unexplained pneumonia. As of April 6th 2020, LTCF residents aged 70 years and older who displayed symptoms (fever (38 degrees Celsius) and cough and/or dyspnoea) were tested for COVID-19 when this was necessary for their care or treatment, regardless of their level of illness. During our study period, testing was only performed in residents with symptoms, meaning no routine testing or testing for asymptomatic cases was done. We have adjusted this part of the manuscript to accurately reflect this:*

*“During our study period, testing was only performed in individuals with symptoms, meaning no routine testing or testing for asymptomatic cases was done.” (lines 107-108)*

**Reviewer 2 Minor Comment 4:** Line 100: I assume these were SARS-CoV-2 antigen assays? Please specify and be precise. Sample collection by the LTCF or sample testing? LTCF can’t do PCR testing.

*We agree that the type of test should be specified. We have added the following to the manuscript:*

*‘Nasopharyngeal and throat swabs from COVID-19 suspected LTCF residents were collected by LTCF staff and sent to the microbiological lab for PCR-testing.’ (lines 116-117)*

**Reviewer 2 Minor Comment 5:** Line 181-182: Not clear what was done for 959 cases (30%) and what happened to the remaining 70%?

*We thank the reviewer for this comment. We agree that this part is still a bit unclear. What we were trying to convey was that for 30% of all cases we assumed an empty cell in our registry meant that the specific symptom was not present. For the rest of the cases (the remaining 70%) such an assumption was not necessary, as all symptoms were clearly registered as a ‘yes’ when they were present or a ‘no’ when they were not present. Therefore, we have adjusted this part of the manuscript to better reflect this:*

*‘Cases for which no registry was made (i.e. lacking a ‘yes’ or ‘no’ for a symptom) were recoded to a ‘no’. This was done for 959 cases (30%). The remaining 70% of cases did not have any empty registries and therefore did not require recoding.’ (lines 160-163)*

**Reviewer 2 Minor Comment 6:** Line 190-195: I suggest to omit “For some symptoms…on other variables” and add a foot note to the respective table.

*In the previous round of revisions, reviewer 1 indicated that only a note was insufficient. We agree with reviewer 1 that only a note would be too brief and might be unclear to the reader. Therefore, we would like to keep this part of the manuscript the same.*

**Reviewer 2 Minor Comment 7:** Line 223-225: you may would like to say something about Tables S1 and S2. Why do you show these tables, what should the reader catch from the data?

*We thank the reviewer for this comment. We briefly address the characters of our study population with regards to underlying comorbidities. Although these comorbidities were not included in further analyses, we would like to give the reader an indication of how our study population was characterized. We therefore included tables S1 and S2 should the reviewer/reader be interested in examining these data. We think that the following adjusted section better clarifies this:*

*‘The most reported comorbidities in COVID-19 positive residents were cardiovascular disease, dementia/Alzheimer, and diabetes. These characteristics of our study population are shown in the supplemental S1 Table. Descriptive statistics on cases without a test result are shown in the supplemental S2 Table. Due to the high number of missing data for comorbidities, these data were not included in further analyses.’ (lines 202-207)*

**Reviewer 2 Minor Comment 8:** Line 286: ‘relevant’ for what?

*Relevant may have been too ambiguous. We therefore have adjusted this section to the following:*

*“This large study, which included LTCF residents and staff during the first COVID-19 wave in a Dutch region, suggests that subfebrility and fatigue may also be symptoms suggestive of COVID-19 in LTCF residents.”(lines 266-268)*

**Reviewer 2 Minor Comment 9:** Line 288: Does that mean that non-associated symptoms are not typical for COVID-19 (i.e. runny nose)?

*We thank the reviewer for this critical remark. We do not claim that symptoms such as a runny nose are not typical for COVID-19, but other symptoms such as fever, cough, and a loss of taste and/or smell seem to be more discriminative to a COVID-19 diagnosis. Coincidentally, these symptoms also appear to be associated to a COVID-19 diagnosis in LTCF staff in our dataset.*

**Reviewer 2 Minor Comment 10:** Line 297: “but our findings suggest aspecific symptoms to be more suggestive for COVID-19 test in residents” : I don’t understand what the authors are trying to say here. Please rephrase.

*We agree that this part was somewhat unclear.* *Our findings suggest that aspecific symptoms are more discriminative for a COVID-19 diagnosis than symptoms which are normally attributed to COVID-19 in LTCF residents, such as a loss of taste and/or smell, fever and cough. To better reflect this, we have adjusted this section as followed:*

*“Although fever, cough, and dyspnea have been reported frequently in our study, our findings suggest that aspecific symptoms are more discriminative for a COVID-19 diagnosis than symptoms which are normally attributed to COVID-19 in LTCF residents, such as a loss of taste and/or smell, fever and cough.” (lines 277-281)*

**Reviewer 2 Minor Comment 11:** Line 365-366: A large sample size does not mean that conclusions can be generalized to LTCFs in other regions or countries. The population of LTCFs in other regions/countries might be very different from those in the South of Limburg.

*We agree with the reviewer and have omitted this part of the manuscript.*

**Reviewer 2 Minor Comment 12:** Line 366-373: I suggest to omit these lines from the discussion since it is (or must be) clear from your methods that all specimens were tested by the same PCR platform and laboratory. I think you can say that it is likely that the CFR is somewhere between the estimated CFR without and estimated CFR with the suspected deaths, but I don’t think it is correct that adding suspected deaths to your analysis have improved the accuracy of the estimate.

*We thank the reviewer for their comment. Although it should be clear from the methods section that all specimens were tested by the same PCR platform and laboratory, this is still a strength of the study. Therefore, we suggest leaving this section as is. However, we do agree with the reviewer that the accuracy did not necessarily improve. Therefore, we have decided to omit ‘Furthermore, by taking into the account the suspected deaths which have most likely been caused by COVID-19, we were able to estimate a more accurate representation of the CFR in this vulnerable population’ from the manuscript.*

**Reviewer 2 Minor Comment 13:** Line 395-397: you need to show the data if you make statements like “most test were done in connection to the onset of symptoms” and “so reasonable compatibility might be expected”. Both phrases are not correct English so need to be rephrased.

*We agree with the reviewer and have decided to omit this part of our manuscript.*

**Reviewer 2 Minor Comment 14:** Line 400: add reference for limited Ct-value differences among variants. Is this the scientific consensus? I thought studies had found significant differences in the viral load between SARS-CoV-2 variants.

*We kindly refer the reviewer to Reviewer 2 Minor Comment 15 in which we also address this comment.*

**Reviewer 2 Minor Comment 15:** Line 400-402: The authors refer here to their own study showing a 1000-fold higher viral load for Delta compared to non-VOC strains. How can you say that the variance in viral load between variants seem to be limited?! What do other studies find? And how can you make a prediction on this topic for the future?

*The reviewer refers to a ‘1000-fold higher viral load for Delta compared to non-VOC strains’. However, this number is based on studies in which the sample size was limited. In our referred study, a total of 16,185 samples were compared. Within a (WGS-confirmed) subset of these 16,185 samples, viral load was found to be significantly higher in the delta variant compared to the non-VOC or alpha variant, though not as high as a 1000-fold (about 7-fold and about 4-fold respectively). Other studies such as a study by Teyssou et al. (DOI:https://doi.org/10.1016/j.jinf.2021.08.027) also found significant differences between the different variants, although not exceeding a 10-fold increase (when examining the ORF1ab target gene of the wildtype variant and the Delta variant). Because the reported difference in viral load between these studies are significantly lower than previously reported by Li et al. (a 1000-fold increase), we phrased the difference in viral load to be ‘limited’.*

*However, in hindsight, we agree with the reviewer that a 4-fold is too much of an increase to call ‘limited’. We have added two additional references and have adjusted this part of the manuscript to the following:*

*“One should note that the results discussed in this study are on the wildtype Wuhan strain of SARS-CoV-2. Ct-values seem to differ per virus variant; higher viral loads have been observed in the alpha, beta, and delta variants of SARS-CoV-2 compared to the wildtype variant [60, 61], although significantly lower than previously reported [62]. It is likely that (future) different variants will have different levels of viral load compared to the wildtype variant examined in this study. However, the relationship between the level of viral load and the appearance of symptoms may still be applicable. Future studies are needed to verify this.” (lines 376-383)*

**Reviewer 2 Minor Comment 16:** Line 403-404: Sorry, I don’t understand what the authors mean by “as symptoms, which a person may have in COVID-19 infections following the first COVID-19 wave.”

*We agree with the reviewer this was phrased unclearly. We have adjusted this to the following:*

*“Furthermore, the effect of vaccination and previous infection may influence the viral load, as well as which symptoms a person may have in future infections.”(lines 383-385)*

**Reviewer 2 Minor Comment 17:** Line 383: explain “prepared studies”.

*Using the term ‘prepared’ may have been unclear. Because this study was set up ad hoc, we were not able to address certain challenges which likely would not have been a problem if the study was not conducted in a time of international crisis. To clarify this, we have changed the term ‘prepared’ with ‘planned’.*

**Reviewer 2 Minor Comment 18:** Line 395: you could have included days since symptom onset on the x-axis of Figure 3.

*We thank the reviewer for this suggestion. However, because data collection was hectic at the time, days since symptoms was not always registered and would have resulted in too many missing data to use as part of this figure.*

**Reviewer 2 Minor Comment 18:** Line 403: do symptoms have an effect on viral load or vice versa?

*We hypothesise that viral load has an effect on which symptoms arise. However, in this part of the manuscript, we propose that the effect of vaccination or a previous infection may influence the viral load found in future infections, as well as which symptoms appear in a future infection.*

**Reviewer 2 Minor Comment 19:** Figure 2: y-axis: “COVID-19” instead of “COVID”.

*We have adjusted this per reviewer feedback.*
